# Feasibility of multi-sector policy measures that create activity-friendly environments for children: results of a Delphi study

**DOI:** 10.1186/1748-5908-6-128

**Published:** 2011-12-15

**Authors:** Marie-Jeanne Aarts, Albertine J Schuit, Ien AM van de Goor, Hans AM van Oers

**Affiliations:** 1 Tilburg University, Tilburg School of Social and Behavioral Sciences, Department Tranzo, Scientific Center for Care and Welfare, PO Box 90153, 5000 LE Tilburg, The Netherlands; 2 National Institute for Public Health and the Environment, Public Health and Health Services Division, PO Box 1, 3720 BA Bilthoven, The Netherlands; 3 VU University Amsterdam, Department of Health Sciences and EMGO Institute for Health and Care Research, De Boelelaan 1105, 1081 HV Amsterdam, The Netherlands

## Abstract

**Background:**

Although multi-sector policy is a promising strategy to create environments that stimulate physical activity among children, little is known about the feasibility of such a multi-sector policy approach. The aims of this study were: to identify a set of tangible (multi-sector) policy measures at the local level that address environmental characteristics related to physical activity among children; and to assess the feasibility of these measures, as perceived by local policy makers.

**Methods:**

In four Dutch municipalities, a Delphi study was conducted among local policy makers of different policy sectors (public health, sports, youth and education, spatial planning/public space, traffic and transportation, and safety). In the first Delphi round, respondents generated a list of possible policy measures addressing three environmental correlates of physical activity among children (social cohesion, accessibility of facilities, and traffic safety). In the second Delphi round, policy makers weighted different feasibility aspects (political feasibility, cultural/community acceptability, technical feasibility, cost feasibility, and legal feasibility) and assessed the feasibility of the policy measures derived from the first round. The third Delphi round was aimed at reaching consensus by feedback of group results. Finally, one overall feasibility score was calculated for each policy measure.

**Results:**

Cultural/community acceptability, political feasibility, and cost feasibility were considered most important feasibility aspects. The Delphi studies yielded 16 feasible policy measures aimed at physical and social environmental correlates of physical activity among children. Less drastic policy measures were considered more feasible, whereas environmental policy measures were considered less feasible.

**Conclusions:**

This study showed that the Delphi technique can be a useful tool in reaching consensus about feasible multi-sector policy measures. The study yielded several feasible policy measures aimed at physical and social environmental correlates of physical activity among children and can assist local policy makers in designing multi-sector policies aimed at an activity-friendly environment for children.

## Background

As in many other affluent countries, lack of physical activity among children is a serious problem in the Netherlands [1], and this has several unfavorable health consequences [2-4]. Next to individual characteristics, physical and social environmental characteristics, such as access to recreational facilities, traffic situation, social safety, and social cohesion, are related to children's physical activity behavior such as outdoor play, sports participation, or active commuting to school [5-8].

Creating environments that are attractive and stimulating for children to be physically active seems a promising strategy to increase physical activity among children [8,9]. In their report on promotion of active living in urban environments, the European division of the World Health Organization highlights the role of local governments in creating activity-friendly environments [10]. Policy measures from policy sectors outside the public health domain--for example spatial planning, traffic and transportation, safety, and social affairs--are warranted to create activity-friendly environments for children [11-14]. Recently, several Dutch advisory boards concluded that there is a large potential health gain if national and local governments adopt a multi-sector approach in tackling health problems such as physical inactivity [15].

Although much research has been conducted into the environmental determinants of physical activity among children, less is known about the opportunities for multi-sector policy measures to address these determinants. Swinburn *et al*. argue that 'evidence is not sufficient by itself to guide appropriate decision making' [16]. Values, policy context, resources and habits, and tradition play a role in the political decision-making process [13,17], and the perceived feasibility of policy measures affects the chance that policy measures will be implemented [18]. Snowdon *et al*. distinguish five different aspects of feasibility: political feasibility, cultural/community acceptability, technical feasibility, cost feasibility, and legal feasibility [19,20]. These feasibility aspects largely correspond to the findings from the PorGrow project, which identifies six groups of criteria for assessing feasibility: societal benefits, additional health benefits, efficacy, economic cost to public sector, economic cost to individuals, economic costs to commercial sector, practical feasibility, and social acceptability [21]. Swinburn *et al*. further mention the availability of a trained work force, the strength of the organizations, networks, systems and leaderships involved, and existing pilot or demonstration programs as possible factors in determining the feasibility of policy initiatives [16].

The aims of this study are: to identify a set of tangible (multi-sector) policy measures at the local level that address environmental characteristics related to physical activity among children; and to assess the feasibility of these measures, as perceived by local policy makers. This research yields locally relevant recommendations that can assist local policy makers in developing multi-sector policies that create activity-friendly environments for children.

## Methods

### Study setting

The study was conducted in four medium-sized Dutch municipalities that were participating in a large-scale research project described in more detail elsewhere [22]. To guarantee complete anonymity of the respondents in the study, city names are blinded throughout the text. Table 1 summarizes the main characteristics of the cities that were enrolled. Despite the fact that municipality D was somewhat smaller compared to the other municipalities, the municipalities were similar regarding the composition of their population.

**Table 1:** Population characteristics of municipalities included in the study^a^

Municipality	A	B	C	D	The Netherlands
Total number of inhabitants	201,259	170,349	135,648	77,450	16,357,992
Degree of urbanization (number of inhabitants per km^2^)	1,716	1,344	1,606	727	394
Percentage inhabitants aged 0-14 years (%)	16.7	17.3	17.2	17.6	18.1
Percentage Western immigrants (%)^b^	8.2	10.0	8.6	8.7	8.8
Percentage non-Western immigrants (%)^c^	13.4	10.2	9.9	11.9	10.6
Number of municipal employees	1,915	2,189	1,430	679	NA

^a ^Characteristics per 1 January 2007 (start date of the research project). All data derived from CBS Statline [1] or the municipal organization (for number of employees).

^b ^Immigrants are defined as persons with at least one parent born in a foreign country. Western immigrants are all immigrants from Europe (with exception of Turkey), North-America, Oceania or Indonesia or Japan.

^c ^Immigrants are defined as persons with at least one parent born in a foreign country. Non-western immigrants are all immigrants from Turkey, Africa, Latin-America or Asia (with exception of Indonesia and Japan).

NA = Not applicable.

### Delphi method

The Delphi method is a well-founded method for reaching consensus among stakeholders in complex (policy) problems [23] and has been widely used in the field of health policies related to obesity [24-27]. Within the Delphi method, respondents are provided with the opportunity to adjust their opinion based on group's mean or median scores in two or more consecutive Delphi rounds and the procedure stops when consensus is reached or response rates decrease [28]. In this study, four separate Delphi studies were conducted (one in each municipality), to provide the municipalities with locally relevant results, which increases the applicability of the research in the municipal policy development process.

### Participants

In the Netherlands, three levels of government exist: national, regional, and local/municipal. The municipal government consists of a bureaucratic system staffed by policy officers, a political level of alderman and mayor, and a municipal council. The policy officers support the aldermen and mayor in administrating the municipality. The members of the municipal council supervise the aldermen and mayor and hold power of decision. Whereas the aldermen and municipal council members are re-elected every four years, the pool of policy officers remains more stable over time. Respondents in this study were chosen from the policy officers because they are best informed about the content of the policies within their sector. Respondents were selected by means of 'snowball sampling', starting with existing contacts with policy officers in the public health domain, who referred to their colleagues from other policy sectors. In each municipality, six policy sectors (public health, sports, youth and education, spatial planning/public space, traffic and transportation, and safety) were invited for participation because of the potential influence on the environmental determinants of children's physical activity [18]. On the respondents' initiative, an additional policy sector was invited in municipality B (environmental affairs), municipality C (economic affairs), and municipality D (play facilities). In addition to policy officers from the municipal organization, the regular policy advisors from the Regional Public Health Services for each municipality were invited to participate in the Delphi study of their particular municipality. In order to prevent overrepresentation of particular policy sectors, a maximum of two respondents per policy sector within each municipality was set. In total, 36 respondents were invited for participation.

### First Delphi round: brainstorm with policy makers

The first Delphi round took place at the venue of the city hall and took approximately 90 minutes. The main results of our survey on environmental correlates of physical activity among children (conducted in the participating municipalities) were presented by the principal researcher of the project [5] and discussed in relation to the state of the art knowledge from scientific reviews [6,29]. During this discussion, it emerged that physical as well as social neighborhood characteristics are related to different components of children's physical activity behavior (*e.g*., outdoor play, active commuting to school, sports participation). Based on these insights, social cohesion, accessibility of facilities, and traffic safety were considered important environmental correlates of different aspects of children's physical activity [5,6,29] that are affected by policy measures of different policy sectors (*e.g*., youth and education, spatial planning, traffic, and transportation). Participants were asked to identify possible municipal policy measures that address these three determinants in a plenary brainstorm chaired by a professional discussion leader (20 minutes per determinant, or one hour for the total brainstorm). Respondents were explicitly asked not to consider the feasibility of policy measures during this first round. At the end of the first round, respondents summarized the identified policy measures from this round by compiling a list of at least four policy measures per determinant, and these were further explored during the second Delphi round. The discussion leader made certain that there was a common understanding of the exact content of the policy measures among the participants of the Delphi meetings (for a detailed description of each policy measure see Additional file 1).

### Second Delphi round: feasibility of policy measures

The second Delphi round (which took approximately two hours) followed immediately after the first Delphi round and took place in the venue of the city hall as well. Five feasibility aspects derived from the literature (political feasibility, cultural/community acceptability, technical feasibility, cost feasibility, and legal feasibility [19,20]) were briefly introduced. Thereupon, each respondent individually weighted the general importance of each of these different aspects of feasibility in the policy development process by dividing 100 points over the five feasibility aspects. Subsequently, each respondent was provided with a printed questionnaire and scored the policy measures derived from the first Delphi round on the five aspects of feasibility (seven-point Likert-type scale, higher scores indicated higher feasibility). These questionnaires were completed and handed in during the meeting at the city hall.

### Third Delphi round: group consensus

The aim of the third Delphi round was to develop group consensus, and this round consisted of a printed questionnaire sent to the respondents by post approximately one week after the first and second Delhi round. Respondents were provided with their own scores, as well as the median group scores from the second Delphi round and were asked to re-evaluate their individual feasibility scores. Respondents unable to attend the first and second Delphi round were invited to evaluate the feasibility of the policy measures during the third Delphi round as well. These respondents were asked to first weigh the five aspects of feasibility (similar to the other respondents) and were provided with the median group scores from the second Delphi round as well. Detailed descriptions of each policy measure (including practical examples mentioned during the discussion meetings) were provided for each respondent (see also Additional file 1). Respondents could return the completed questionnaire of the third Delphi round by an enclosed prepaid envelope.

### Data analysis

By multiplying the individual weighting scores by the feasibility scores on each feasibility aspect and summing the five feasibility scores for each policy measure, one overall (weighted) feasibility score per policy measure was calculated for each respondent for the second and third Delphi round separately. The median weighted overall feasibility score was then computed per policy measure for each municipality for the second and third Delphi round separately. In addition, the standard deviation (SD) was calculated as an indicator for consensus within each municipality for the second and third Delphi round separately (higher SD scores indicate less consensus). Two respondents had missing values on their weighting scores, and therefore these were imputed by the average weighting scores of the other respondents within the same municipality. Three respondents that participated in the first and second Delphi round did not return or had missing values on the questionnaire during the third Delphi round. In those cases, the missing scores on the third Delphi round were replaced by the respondent's scores from the second round.

Policy measures were considered 'consistently feasible' if they met the following conditions in the third Delphi round: weighted median overall feasibility score ≥ 5.00; standard deviation ≤ 1.00; and the minimum overall feasibility score given by any individual respondent within that municipality ≥ 3.50. Policy measures were considered 'consistently less feasible' if they met the following conditions: weighted median overall feasibility score ≤ 4.00; and standard deviation ≤ 1.00 in the third Delphi round. These criteria were chosen as natural cut off points based on the scales used and data obtained. To further compare results across municipalities, each policy measure was classified into one or more of the following categories: communicative policy measures such as health education and advertisements; juridical policy measures such as laws and prohibitions; economic policy measures such as subsidies, grants, charges, and taxes; and environmental policy measures such as changes in facilities, infrastructure, or neighborhood design. All policy measures were classified by two authors independently, and in case of inconsistencies, consensus on classification was reached by discussion.

## Results

**Figure 1:**
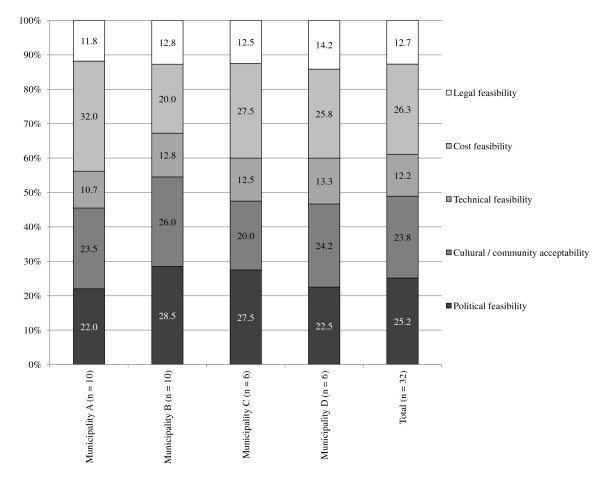
**Perceived importance of feasibility aspects per municipality**.

**Figure 2:**
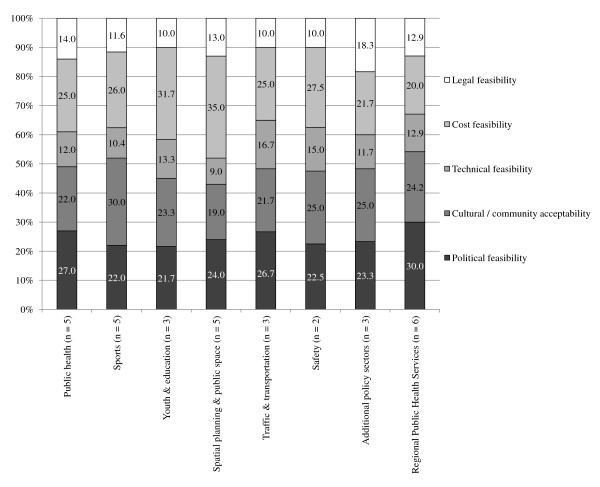
**Perceived importance of feasibility aspects per policy sector**.

The overall response rate was 72.2% (range among municipalities 50.0% to 90.0%) in the first and second Delphi round and 88.9% (range 75.0% to 100.0%) in the third Delphi round (Table 2). Figures 1 and 2 show that the importance respondents assign to the different aspects of feasibility are roughly the same per municipality and per policy sector: legal feasibility and technical feasibility were considered less important and cultural/community acceptability, political feasibility, and cost feasibility were considered of greater importance. Furthermore, respondents indicated that the three most important feasibility aspects were highly interconnected. According to the respondents, political feasibility is influenced by politicians' perceptions of community acceptability, due to electoral considerations. The political feasibility on its turn defines the financial resources that are reserved for certain policies and hence influences the cost feasibility.

**Table 2:** Participants and response rates per municipality in the different Delphi rounds

Municipality	First and second Delphi round			Third Delphi round		
	**Invited**	**Participated**	**Response**	**Invited**	**Participated**	**Response**

A	10 (6 m, 4 f)	9 (5 m, 4 f)	90.0%	10 (6 m, 4 f)	10 (6 m, 4 f)	100.0%
B	10 (2 m, 8 f)	8 (1 m, 7 f)	80.0%	10 (2 m, 8 f)	9 (2 m, 7 f)	90.0%
C	8 (4 m, 4 f)	5 (2 m, 3 f)	62.5%	8 (4 m, 4 f)	6 (4 m, 2 f)	75.0%
D	8 (4 m, 4 f)	4 (2 m, 2 f)	50.0%	8 (4 m, 4 f)	7 (4 m, 3 f)	87.5%
Total	36 (16 m, 20 f)	26 (10 m, 16 f)	72.2%	36 (16 m, 20 f)	32 (16 m, 16 f)	88.9%

Table 3 shows all policy measures aimed at increasing social cohesion, accessibility of facilities and traffic safety that were put forward by the respondents of the four municipalities (a detailed description of each policy measure is given in Additional file 1). The scores from the third Delphi round are presented in Table 3 (data from the second Delphi are not shown, but can be obtained from the author on request). All municipalities showed an increase in perceived overall feasibility and consensus from the second to third Delphi round for the majority of policy measures, except for municipality B where consensus decreased from the second to the third Delphi round for the majority of policy measures.

**Table 3:** Perceived feasibility of possible policy measures addressing environmental determinants of physical activity among children

Policy measures developed in the first Delphi round	Type of policy measure	Perceived overall feasibility (weighted median score)^a^	Consensus (SD)	Range
Municipality A: social cohesion				

**1. Multi-use of school yards^b^**	**Juridical/environmental**	**5.08**	**0.75**	**4.00-6.37**
2. Subsidy for citizens' initiatives to increase social cohesion^b^	Economic	5.05	0.80	3.10-6.30
3. Democratic decision process when implementing new neighborhood facilities	Juridical	4.65	0.65	4.00-6.30
4. Stimulate/oblige parents to choose primary school within own neighborhood^b^	Communicative/juridical	4.30	1.26	1.00-5.47
5. Spatial planning that enhances daily encounters^b^	Environmental	4.10	0.95	2.90-6.20

Municipality A: accessibility of facilities				

**6. Attractive (walking) routes for children^b^**	**Environmental**	**5.19**	**0.83**	**3.60-7.00**
**7. Informal play facilities (fallow lands, sand hills)**	**Juridical/environmental**	**5.01**	**0.81**	**3.80-6.50**
8. Multi-use of vacant parking places^b^	Juridical/environmental	4.58	0.80	3.00-5.30
9. Outdoor exercise facilities for adults (role models)	Environmental	4.50	0.74	3.00-5.50
10. Increase economic accessibility of sport facilities	Economic	4.10	0.65	3.50-5.70

Municipality A: traffic safety				

11. Local Safety Label for primary schools	Communicative	5.25	1.02	3.70-6.70
12. Fencing off streets for outdoor play	Juridical/environmental	5.25	1.41	2.00-6.04
**13. Enhance responsibility of school boards and parents for traffic safety^b^**	**Communicative**	**5.20**	**0.73**	**3.60-6.00**
14. Car-free/low-traffic school zones during peak hours^c^	Juridical/environmental	4.43	0.70	3.20-5.15

Municipality B: social cohesion				

15. Use major changes in neighborhoods to increase social cohesion	Communicative	5.43	0.90	3.00-6.20
16. Stimulate initiatives of citizens to increase social cohesion^b^	Economic/communicative	5.22	1.03	2.80-6.80
17. Multi-use of school yards^b^	Juridical/environmental	5.05	1.51	1.30-7.00
18. Increase social cohesion by business licensing requirements	Juridical	4.61	0.70	3.80-6.10

Municipality B: accessibility of facilities				

19. Attractive (walking) routes for children^b^	Environmental	5.51	1.09	2.80-7.00
20. Multi-use of vacant parking places^b^	Juridical/environmental	4.48	0.64	3.90-5.63
*21. Dispersal of play facilities over the neighborhood*	*Environmental*	*3.83*	*0.74*	*2.90-5.50*
22. Car free neighborhoods	Environmental	3.35	1.11	2.30-5.80

Municipality B: traffic safety				

**23. Supervised active commuting to school**	**Communicative**	**5.68**	**0.99**	**3.90-6.85**
24. Increase awareness for active commuting to school	Communicative	4.90	1.04	2.80-6.35
25. School zones that discourage cars^c^	Juridical/environmental	4.40	0.85	2.70-5.60
26. Infrastructural facilities that help children reach popular destinations	Environmental	4.20	0.81	2.70-4.95

Municipality C: social cohesion				

**27. Fencing off streets for outdoor play^b^**	**Juridical/environmental**	**5.05**	**0.83**	**4.05-6.53**
28. Maintain play function of play facilities for children	Juridical	4.55	0.40	4.00-5.10
*29. Stimulate/oblige parents to choose primary school within own neighborhood^b^*	*Communicative/juridical*	*3.70*	*0.54*	*3.15-4.50*
30. Improve neighborhood's population composition	Juridical/economical	2.85	1.55	1.75-6.00

Municipality C: accessibility of facilities				

31. Parking policies that stimulate active transportation	Environmental	4.90	0.64	4.00-5.80
32. Attract facilities in the neighborhood by adjusting the municipal zoning plan	Juridical	4.90	0.44	4.20-5.45
*33. Physical education facilities in the direct surroundings of the school*	*Environmental*	*3.80*	*0.35*	*3.60-4.55*
*34. Dependences of well-known (professional) sport clubs in the neighborhood*	*Environmental*	*3.00*	*0.76*	*2.60-4.75*

Municipality C: traffic safety				

**35. Communication around active commuting**	**Communicative**	**5.60**	**0.46**	**5.20-6.65**
**36. Traffic education for children at primary schools**	**Communicative**	**5.40**	**0.69**	**4.40-6.45**
37. Attractive routes for recreation (bicycling, skating)	Environmental	4.30	0.57	3.80-5.20
*38. Improve public transportation supply*	*Environmental*	*3.85*	*0.89*	*2.80-5.55*

Municipality D: social cohesion				

**39. Assign a part of the neighborhood maintenance budget to citizens**	**Economic**	**5.50**	**0.76**	**4.00-6.06**
**40. Organizing agreements with local actors about neighborhood activities**	**Juridical/communicative**	**5.40**	**0.48**	**4.75-6.20**
**41. Assign part of the budget for neighborhood activities to local actors**	**Economic**	**5.30**	**0.59**	**4.10-5.70**
**42. Neighborhood agreements that increases the feeling of social safety**	**Juridical/communicative**	**5.15**	**0.68**	**4.80-6.50**
**43. Spatial planning that enhances daily encounters^b^**	**Environmental**	**5.10**	**0.53**	**4.40-5.90**

Municipality D: accessibility of facilities				

**44. Safety Impact Assessment for all sport facilities**	**Juridical/communicative**	**5.80**	**0.91**	**4.30-6.80**
45. Physical infrastructure to increase the accessibility of sport facilities	Environmental	5.40	1.15	2.57-5.75
46. Spatial planning that fits the needs of different target groups (youth, elderly)	Environmental	4.90	0.74	3.50-5.70
47. Location of sport facilities (easily accessible from the neighborhood)	Environmental/juridical	4.45	0.69	3.80-5.70

Municipality D: traffic safety				

**48. Provide users of facilities with information to enhance traffic safety^b^**	**Communicative**	**5.75**	**0.27**	**5.40-6.10**
**49. Couple maximum traffic speeds to standard street types**	**Juridical**	**5.45**	**0.51**	**4.40-5.90**
50. Car-free/low-traffic school zones^c^	Juridical/environmental	4.50	0.57	3.45-5.30
51. Deregulation of traffic situations	Juridical/environmental	4.30	0.52	3.90-5.37

^a ^Within each municipality and within each determinant, policy measures are sorted descending on weighted overall feasibility score during the third Delphi round. Consistently feasible policy measures are represented in bold font, consistently less feasible policy measures are represented in italic font.

^b ^Policy measures that were put forward in two municipalities.

^c ^Policy measures that were put forward in three municipalities.

Although some policy measures could have beneficial effects on more than one of the three environmental characteristics, overall, from the 16 policy measures that were consistently feasible, seven were aimed at improving social cohesion, three were aimed at improving the accessibility of facilities, and six were aimed at improving traffic safety. From the five consistently less feasible policy measures, one was aimed at improving social cohesion, three were aimed at improving accessibility of facilities, and one was aimed at improving traffic safety.

Although some policy measures could be classified into more than one category, overall, from the 16 policy measures that were consistently feasible, five measures were predominantly communicative, seven were predominantly juridical, two were predominantly economical, and two were predominantly aimed at changes in the environment. From the five consistently less feasible policy measures, one was communicative/juridical, whereas four were predominantly aimed at changes in the environment.

## Discussion

The aims of this study were: to identify a set of tangible (multi-sector) policy measures at the local level that address environmental characteristics related to physical activity among children; and to assess the feasibility of these measures, as perceived by local policy makers. In order to achieve this, a three-staged Delphi study was performed in each of the participating municipalities. The results of these three stages will be discussed in a chronological order below. Thereafter, a comparison with previous research is made, and the strengths and limitation of this study are discussed.

During the first Delhi round, several concrete policy measures aimed at social cohesion, accessibility of facilities, and traffic safety were derived. The first Delphi round further showed that cultural/community acceptability, political feasibility, and cost feasibility were considered of greatest importance in evaluating the feasibility of local policy measures

The objective of the Delphi technique is to reach consensus among participants, a goal that was met in three out of four municipalities in this study while passing through the Delphi protocol. Although no direct cause for the absence of increase in consensus in municipality B could be distinguished, the Delphi technique also generated many feasible policy measures in this municipality. The increase in feasibility from the second to third Delphi round observed for many policy measures might be explained by the fact that respondents initially were unfamiliar with the concept of creating activity-friendly environments, and therefore perceived such policy measures to be less feasible at first. As they might have become more familiar with the concept of activity-friendly environments during the third Delphi round, this might have increased the perceived feasibility.

Finally, there were more policy measures classified as consistently feasible than as consistently less feasible in the third Delphi round. This might reflect the respondents' tendency to think in a constructive way about possible policy measures. In municipality D, many policy measures could be marked as consistently feasible. This could be partly due to the fact that in this municipality, the brainstorm tended to focus on policy measures that already existed in some neighborhood(s), but could be broadened to other neighborhoods as well. In municipality C on the contrary, the discussion focused more on theoretically possible policy measures, which might explain why in this municipality more policy measures were classified as consistently less feasible.

### Comparison with previous research

In line with the findings of this study, the importance of economic and political factors was also mentioned by municipal employees in previous research [18,30]. Although respondents initially were less familiar with the possibilities to improve social cohesion within their municipality, during the Delphi process they became aware of several feasible policy measures that address this determinant of children's physical activity. Policy measures aimed at improving traffic safety were also perceived as feasible. However, policy measures aimed at improving accessibility of facilities were considered less feasible, probably because this requires drastic modifications in the built environment. Policy measures that have a more authoritative character (*e.g*., obliging parents to choose a primary school within their own neighborhood) were also rated less feasible. These findings are in line with the cross-national results from the European PorGrow project, gathered among a broad spectrum of governmental and non-governmental stakeholders. The PorGrow results indicate that although the need for an integrated approach aimed at environmental changes is recognized, less drastic policy options aimed at education and information for parents and children are generally ranked highest [31]. The PorGrow results further show that economic policy measures such as subsidies and taxes are given low appraisal scores [31].

In their Delphi study among Dutch experts on opportunities for monetary incentives to stimulate healthy eating, Waterlander *et al*. have shown that experts tend to rate policy options outside their own area of responsibility more positively [26]. Although respondents did emphasize the own responsibility of parents for their child's activity behavior, there were no indications that respondents from any policy sector tended to pass the responsibility to other policy sectors in the present study. This was probably due to the fact that the respondents were mostly colleagues within the same organization and already discussed the policy options together during the first Delphi round.

### Strengths and limitations of this study

Although the aim of this study was to cover roughly the same policy sectors within each municipality, the exact job description of the respondents did vary between municipalities, and diversity of respondents between municipalities could not be completely eliminated. The fact that this study was aimed at municipality-specific recommendations somewhat limits the possibilities to generalize the results. However, some of the findings (such as the fact that cultural/community acceptability, political feasibility, and cost feasibility were consistently considered of greatest importance) can be generalized to other settings as well. The use of a multi-criteria mapping technique--in which respondents have the opportunity to bring up policy options themselves and to weigh the different evaluation criteria--is a useful strategy to provide respondents with adequate freedom of expression, but nevertheless retain the possibility to compare results across municipalities or countries [21,32]. In addition to assessing the feasibility of different policy measures by assigning scores on Likert-type scales, ranking policy measures could further stimulate participants to single out the different policy alternatives [24,26,28]. Although this study provided the respondents with information on which environmental characteristics could possibly affect children's physical activity behavior during the first Delphi round, no detailed information was available on the (theoretical) effectiveness of the proposed policy measures. Calculating the potential health gains of different policy measures could be of great value because this could help to persuade policy makers to seriously consider the implementation of less feasible, but possibly more effective policy alternatives as well. Future research should evaluate if local policy makers themselves see the Delphi technique as a valuable tool in the development of multi-sector policy measures aimed at health promotion at the local level and if it facilitates their actual adoption and implementation. It would also be interesting to study whether elected officials have the same view on feasibility of the identified policy options identified by policy officers in this study.

## Conclusions

This study showed that the Delphi technique can be a useful tool in identifying feasible multi-sector policy measures aimed at creating activity-friendly environments for children at the local level. Cost feasibility, cultural/community acceptability, and political feasibility are of great importance in evaluating the feasibility of local policy measures. Less drastic policy measures were considered more feasible, whereas environmental policy measures were considered less feasible. Therefore, it is of crucial importance to convince policy makers of the effectiveness of environmental policy measures aimed at stimulating physical activity among children and to persuade policy makers to seriously consider the implementation of less feasible, but possibly more effective policy alternatives as well.

## Competing interests

The authors declare that they have no competing interests.

## Authors' contributions

MJA conducted the Delphi studies, performed the analyses and drafted the manuscript. AJS, LAMvdG and JAMvO supervised the data collection and analysis, contributed to the interpretation of the results and reviewed the manuscript. All authors read and approved the final manuscript.

## Supplementary Material

Additional file 1 **Detailed description of policy measures derived from the first Delphi round**. Detailed description of policy measures derived from the first Delphi round. Click here for file
