# Supplementary material for: Feasibility of multi-sector policy measures that create activity-friendly environments for children: results of a Delphi study
Source: Implement Sci. 2011 Dec 15;6:128. doi: 10.1186/1748-5908-6-128 (PMC3264503; doi:10.1186/1748-5908-6-128)
Supplement: Additional file 1 — Detailed description of policy measures derived from the first Delphi round. Detailed description of policy measures derived from the first Delphi round. [file 1748-5908-6-128-S1.PDF]

Additional file 1
Detailed description of policy measures derived from the first Delphi round

Municipality A: social cohesion

1. Increase multi-use of school yards so that children can play there after school time and in the weekends (e.g. by placement of fences and appointment of key holders).
2. Subsidies for initiatives of citizens to increase social cohesion (e.g. street barbeques, coffee break projects, shared play equipment in the neighborhood).
3. Establish a democratic decision process when implementing new neighborhood facilities so that people get involved in spatial planning issues in their neighborhood and NIMBY effects are overcome.
4. Stimulate or oblige parents to choose a primary school for their children within their own neighborhood (e.g. provide parents with information on the primary schools within their neighborhood or assign primary school based on postal codes of the parents' residence).
5. Enhance daily encounters between people by taking this into account in spatial planning (e.g. front gardens, dog walking areas, benches etc.).

Municipality A: accessibility of facilities

6. Construct attractive (walking) routes for children to popular facilities (e.g. by means colorful, playful street design).
7. Facilitate informal play facilities (e.g. by providing children access to fallow lands, sand hills etc at construction sites).
8. Stimulate multi-use of vacant parking places (e.g. business parking places that are vacant in the weekends) so that they can be used as play grounds.
9. Provide with outdoor exercise facilities for adults, so that they can serve as a role model for children.
10. Increase the economic accessibility of sport facilities (e.g. by subsidies for poor families).

Municipality A: traffic safety

11. Stimulate primary schools to acquire the Local Safety Label.
12. Fence off streets for a couple of hours to create opportunities for safe outdoor play (e.g. every Wednesday afternoon).
13. Stimulate the own responsibility of school boards and parents to maintain traffic safety around their primary school (because often the parents create unsafe situations themselves by fetching and collecting their children by car, and this should be brought under their attention).
14. Create car-free/low-traffic school zones during the peak hours for parents to fetch/collect their children (e.g. by means of a barrier).

Municipality B: social cohesion

15. Use major changes in neighborhoods to increase social cohesion (because often, neighborhood's residents unify when faced with major changes).
16. Stimulate initiatives of citizens to increase social cohesion (e.g. street barbeques, cooking classes, supervised reading clubs for children).
17. Increase multi-use of school yards so that children can play there after school time and in the weekends.
18. Increase social cohesion by business licensing requirements (e.g. businesses are

obliged to connect with the residents in their neighborhood by means of internships etc.).

Municipality B: accessibility of facilities

19. Construct attractive routes for children to popular facilities (e.g. by means colorful, playful street design).
20. Stimulate multi-use of vacant parking places (e.g. business parking places that are vacant in the weekends, can be used as parking places for residents so that neighborhoods become car-free and children can reach play facilities more easily).
21. Disperse several (smaller) play facilities over the neighborhood, instead of one central play facility.
22. Realizing car-free neighborhoods so that children can reach play facilities more easily (e.g. by locating parking places at the borders of existing neighborhoods).

Municipality B: traffic safety

23. Stimulate supervised active commuting to school (e.g. older children or relatives such as grandparents supervise groups of younger children in active commuting to and from school).
24. Increase awareness among parents for active commuting to school by means of long-lasting communication campaigns.
25. Create and sustain school zones that discourage cars (parking policies, one-way streets, police control).
26. Realize infrastructural facilities such as crossing places and viaducts that help children reach popular destinations (such as sport and play facilities).

Municipality C: social cohesion

27. Fence off streets at specific days to create opportunities for safe outdoor play.
28. Maintain play function of play facilities for children (e.g. tackle problems with older youth that hangs around).
29. Stimulate or oblige parents to choose a primary school for their children within their own neighborhood.
30. Increase social cohesion by formulating policies that affect the neighborhood's population composition (e.g. by mixing different socioeconomic and ethnical population groups).

Municipality C: accessibility of facilities

31. Develop parking policies that stimulate active transportation to facilities (e.g. providing bicycle racks at facilities).
32. Attract facilities such as shops, hairdressers and physiotherapists in the neighborhood by adjusting the municipal zoning plan.
33. Provide primary schools with adequate physical education facilities in the direct surroundings of the school, so that they can be reached by foot.
34. Realize dependences of well-known (professional) sport clubs in the neighborhood, to facilitate intake of youth.

Municipality C: traffic safety

35. Expand communication around active transportation such as the initiative "park your car and rent a bike" which is set up to keep the city centre free of cars.
36. Provide traffic education for children at primary schools.
37. Create attractive routes for recreation (bicycling, skating) or create connections from neighborhoods to such routes.

38. In collaboration with higher government (province) improve public transportation supply (e.g. frequency of busses, location of bus stops near primary schools etc.)
**Municipality D: social cohesion**
39. Assign a part of the municipal neighborhood maintenance budget to citizens, so that residents become collectively responsible for the maintenance of their own neighborhood.<br>40. Make organizing agreements with local actors about regular neighborhood activities.<br>41. Assign part of the municipal budget for neighborhood activities to local actors so that they become collectively responsible for organizing these activities.<br>42. Increase social cohesion by making neighborhood agreements that define the tasks and roles of different actors in the neighborhood and increases the feeling of social safety among citizens.<br>43. Enhance daily encounters between people by taking this into account in spatial planning (e.g. front gardens, dog walking areas, benches etc.).
**Municipality D: accessibility of facilities**
44. Oblige/stimulate all sport facilities to conduct a Safety Impact Assessment, so that (traffic) safety in and around sport facilities is increased, which in turn increases the opportunities for children to use these facilities independently.<br>45. Provide physical infrastructure such as bike lanes to increase the accessibility of sport facilities.<br>46. Improve spatial planning in such a way that public spaces fit the needs of different target groups (youth, elderly).<br>47. Instead of placing sport facilities at the city borders, situate them in such as way that they become more easily accessible from the neighborhood.
**Municipality D: traffic safety**
48. Provide users and providers of facilities with information, so that they can enhance the traffic safety around their school, sports club etc. themselves.<br>49. Couple maximum traffic speeds to standard street types (30 km/h in residential neighborhoods, 50 km/h in connecting streets, 60 and 80 km/h in areas surrounding the city).<br>50. Create car-free/low-traffic school zones.<br>51. Deregulation of traffic situations i.e. remove excessive traffic signs and infrastructure to increase alertness among road users.
